# Multi-Step Forming Simulation and Quality Control of Aluminum Alloy Automobile Rear Upper Control Arm

**DOI:** 10.3390/ma15103610

**Published:** 2022-05-18

**Authors:** Xiaogu Chen, Xuedao Shu, Donggang Wang, Sheng Xu, Wei Xiang

**Affiliations:** 1 Department of Mechanical Engineering, School of Mechanical Engineering and Mechanics, Ningbo University, Ningbo 315211, China; chenxiaogu2022@163.com (X.C.); xs@zbti.edu.cn (S.X.); xiangwei@nbu.edu.cn (W.X.); 2 Zhejiang Key Laboratory of Parts Rolling Technology, School of Mechanical Engineering and Mechanics, Ningbo University, Ningbo 315211, China; 3 Department of Mechanical Engineering, Zhejiang Business and Technology Institute, Ningbo 315012, China; wangdonggang@zbti.edu.cn

**Keywords:** rear upper control arm, rolling-forging composite forming process, multi-step, quality control

## Abstract

Aluminum alloy is widely used in automobile parts because of its light weight and good process performance. In view of the complex structure of aluminum alloy automobile rear upper control arms, multiple forming processes and the difficulty in quality control, in this paper, we propose a rolling-forging composite process to produce a rear upper control arm. Based on the reasonable volume distribution of the blank by cross-wedge rolling, multi-step forging was carried out. Finite element simulation of thermomechanically coupled multi-step forming was carried out using DEFORM software. Based on a comparison of the traditional process and the proposed rolling-forging composite forming process, we concluded that the rolling-forging composite process can greatly reduce the material cost and the forming force, resulting in superior product performance. The coarse-grain structure of products at different process temperatures was analyzed by a crystal-phase experiment. The results show that the process temperature of the multi-step process, as well as the heat treatment temperature and time have an important influence on the coarse-grain structure of the product. The optimal preheating temperatures for preforging and final forging dies were determined to be 335 °C and 350 °C, respectively; a preheating temperature of 530 °C and a solution time of 45 min resulted in the least coarse-grain surface structure. The research results provide a theoretical basis for improving the multi-step forming quality of automobile rear upper control arms.

## 1. Introduction

With the development of productivity and technology, the automobile industry has entered a new stage. A variety of new vehicles with low energy consumption, low pollution and good safety have gradually become mainstream products as a result of development in the automobile industry. In order to realize automobile energy savings, emissions reductions and lightweight technology, aluminum alloy has been widely used because of its light weight, high specific strength, good process performance and corrosion resistance [1,2]. Some important parts, such as automobile swing arms, are formed by aluminum alloy die forging [3,4]. However, the aluminum alloy forging technology currently used in China lags behind other developed technologies. Due to the lack of appropriate volume distribution, aluminum alloy materials are seriously wasted when producing complex aluminum alloy forgings, with a material utilization rate of only 20–30%, which considerably increases the production cost of aluminum alloy.

Universities and enterprises all over the world have made efforts to improve the utilization rate of aluminum alloy materials. In some developed countries, forging technology for automobile parts has developed from simple, single-station setups and extrusion to multi-step and multi-forming methods. Countries with developed automobile industries, such as America, Japan and South Korea, were among the first to develop aluminum alloy forging technology. Aluminum alloy forging processes have been widely used to produce automobile control arms [5].

Automobile companies such as SAIC-GM, BAIC Benz, BMW Brilliance, Guangzhou Honda and Geely have also used forging technology to produce aluminum alloy control arms [6]. Bao et al. used roll forging to replace free forging and adopted a multi-step forming process to successfully reduce the forming force of die forging and ensure the consistency of products [7]. Zhou et al. used three steps—roll forging, flattening (bending) and die forging—to form the front suspension lower arm of an Audi A6 automobile so as to improve the material utilization rate and product forming quality [4]. Wei proposed a five-step process of roll forging, flattening, bending, preforging and final forging and verified an improvement in product performance by studying the hot deformation behavior and structural properties of 6082 aluminum alloy rib forgings [8]. However, there are still many unsolved problems; for instance, the material utilization rate is still low, production quality can be improved and the production process is unreasonable. As a thermally sensitive material, the deformation process of aluminum alloy is considerably affected by temperature, and the influence of thermal properties on forming process used in the automobile industry has not been thoroughly studied.

**Figure 1:**
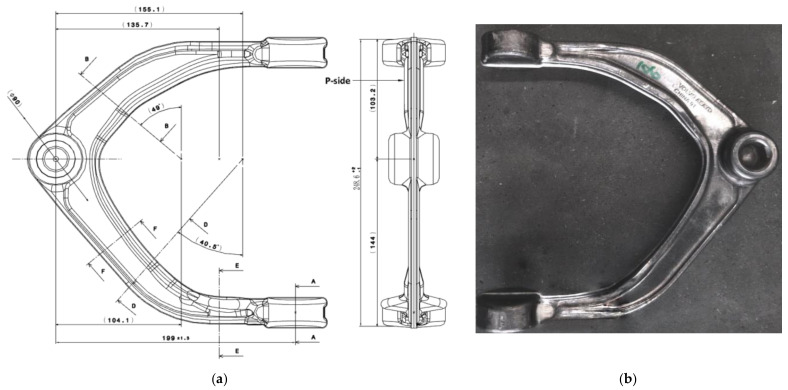
Product schematic. (**a**) Part drawing, (**b**) sample.

In view of the above problems, in this paper, using the rear upper control arm of an automobile as an example, we propose a multi-step forging method employing a forging-rolling composite forming process to improve the material utilization rate of aluminum alloy. Figure 1 shows a schematic drawing and a sample of an aluminum alloy rear upper control arm of an automobile. The product is a ribbed part with a complex shape and complicated features, such as thin web, high rib and bulge. It is difficult to produce qualified forgings for such a product with a simple forging process.

With traditional processing technology, this part is formed with multi-step forming processes such as flattening, bending, preforging and final forging. This forming method has several technical problems, such as high technical difficulty and low material utilization. The process of rolling-forging composite forming proposed in this paper is mainly divided into wedge rolling, bending, flattening, preforging and final forging. The cross-wedge rolling process is used to redistribute the blank to improve the material utilization rate. The material utilization rate can be increased from 20–30% to 50–60%. The forming force of die forging is significantly reduced so as to reduce energy consumption [9,10].

## 2. Solution of Constitutive Model

**Figure 2:**
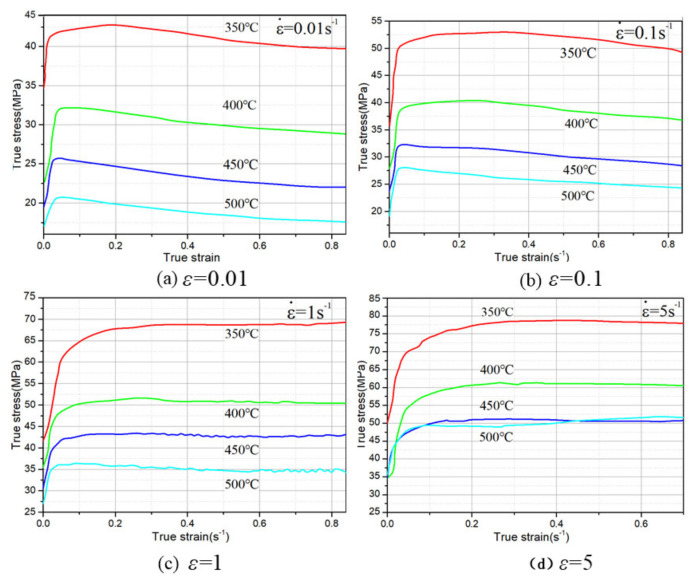
True stress–true strain curves of 6082 aluminum alloy under different deformation conditions.

**Figure 3:**
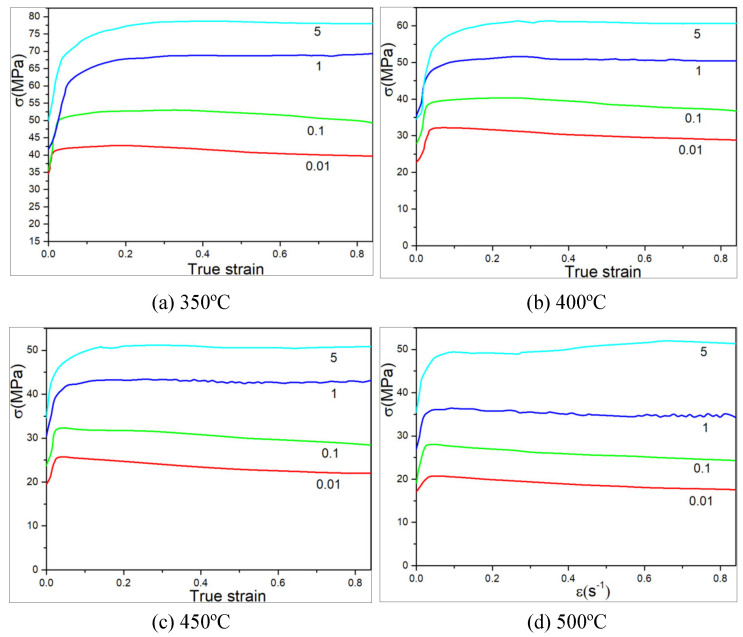
True stress–true strain curves of 6082 aluminum alloy at different preheating temperatures.

Constitutive relationship is the basis for numerical simulation of 6082 aluminum alloy and metal forming process analysis. The true stress–true strain curves of 6082 aluminum alloy at strain rates of 0.01 s^−1^, 0.1 s^−1^, 1 s^−1^ and 5 s^−1^ were obtained in the literature [8,11,12,13,14,15], as shown in Figure 2 and Figure 3.

It can be seen from Figure 2 and Figure 3 that 6082 aluminum alloy is sensitive to temperature and deformation rate. The constitutive equation is established by measuring the flow stress in a range of strain rate and temperature according to the thermal compression experiment [16].

In actual plastic processing, the flow stress, σ, of the material depends on not only the main factors, such as deformation temperature, T; strain, ε; and deformation rate, $\dot{\varepsilon }$, but also other conditions related to materials, such as the material composition, heat treatment system, original grain size and historical deformation. The material and the conditional factors related to the material are represented by constant C, and the functional expression between the flow stress of the material and the deformation temperature, strain, deformation rate and material-related constants is:

(1) $$\sigma \;=f\left(\dot{\varepsilon },\varepsilon ,T,C\right)$$

Once the composition of the material and other conditions related to the material is determined, the flow stress, σ, is only related to the temperature, strain rate and strain during deformation. Moreover, the strain rate is in a functional relationship with strain. Therefore, Equation (1) can be simplified as:

(2) $$\sigma \;=f\left(\dot{\varepsilon },T\right)$$

The effects of deformation temperature and strain rate on flow stress are expressed by Equation (3) [11].

(3) $$\dot{\varepsilon }=Af\left(\sigma \right)\exp\left(\frac{-Q}{RT}\right)$$

In the formula, $\dot{\varepsilon }$ denotes strain rate; *A* is a constant related to the material, representing the flow stress; *f* (σ) is a function related to material stress; *Q* is deformation activation energy related to the material; *R* is a gas constant; *T* is the absolute deformation temperature; and *Q* and *A* are independent of temperature.

The constitutive equation of 6082 aluminum alloy was established by using an Arrhenius constitutive relation and introducing a *Z* parameter to represent the influence of deformation temperature and strain rate on the microstructural deformation of the material. The general form of the Arrhenius constitutive relation is shown in Equation (4) [13]:

(4) $$\dot{\varepsilon }=A{\left[sinh\left(\alpha \sigma \right)\right]}^{n}exp\left(\frac{-Q}{RT}\right)$$

where, α and *n* are the parameter related to the material.

The peak stress, $\sigma_{p}$, of 6082 aluminum alloy under different temperature and deformation rates was calculated by linear regression method. The data of $\sigma_{p}$ are shown in Table 1.

In order to obtain the parameter in and ensure that the relative error is less than 10%, it is necessary to simplify Equation (4). The final result is calculated as $\alpha =0.0276\;\mathrm{MPa},A=4.43\times 10^{10},n=6.735,\;Q=163.792\;\mathrm{KJ}/\mathrm{mol}$. The flow stress constitutive equation of 6082 aluminum alloy applicable to all stress states can be obtained by substituting the α, *A*, *n* and *Q* obtained above into Equation (4):

(5) $$\dot{\varepsilon }=4.43\times 10^{10}{\left[sinh\left(0.0276\sigma \right)\right]}^{6.297}\exp\left(\frac{163792}{RT}\right)$$

In order to verify the correctness of the obtained flow stress model of 6082 aluminum alloy, different deformation conditions are substituted into the hyperbolic sine model to obtain the corresponding flow stress, which is compared with the corrected experimental data. The accuracy of the above model is measured according to the error. Considering the convenience of subsequent comparison, the flow stress is expressed as a function of the Z parameter [17].

Set

(6) $$Z=A{\left[sinh\left(\alpha \sigma \right)\right]}^{n}$$

Then,

(7) $${\left(\frac{Z}{A}\right)}^{\frac{1}{n}}=\sinh\left(\alpha \sigma \right)=\frac{e^{\alpha \sigma }-e^{-\alpha \sigma }}{2}$$

(8) $$e^{2}{}^{\alpha \sigma }-2e^{\alpha \sigma }{\left(\frac{Z}{A}\right)}^{\frac{1}{n}}-1=0$$

(9) $$e^{\alpha \sigma }={\left(\frac{Z}{A}\right)}^{\frac{1}{n}}+\sqrt{{\left(\frac{Z}{A}\right)}^{\frac{2}{n}}+1}$$

Finally,

(10) $$\sigma =\frac{1}{\alpha }\ln\left\{{\left(\frac{Z}{A}\right)}^{\frac{1}{n}}+{\left({\left(\frac{Z}{A}\right)}^{\frac{2}{n}}+1\right)}^{\frac{1}{2}}\right\}\;$$

The value of α, A, n and Q are substituted into Equations (10), and (11) is ultimately determined to calculate the theoretical stress under the corresponding conditions.

(11) $$\sigma_{f}=\frac{1}{0.0276}\ln\left\{{\left(\frac{Z}{4.43\times 10^{10}}\right)}^{\frac{1}{6.297}}+{\left\{{\left(\frac{Z}{4.43\times 10^{10}}\right)}^{\frac{2}{6.297}}+1\right\}}^{\frac{1}{2}}\right\}$$

**Figure 4:**
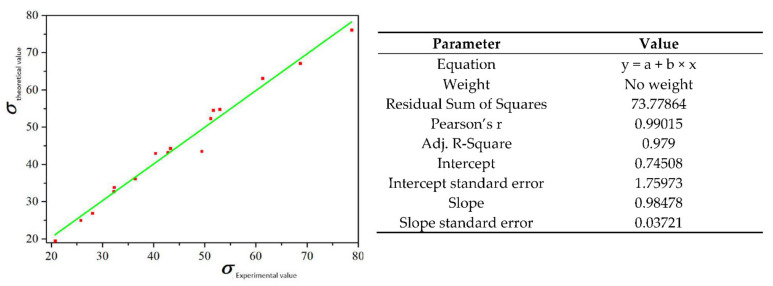
Comparison of theoretical and experimental values.

The peak strain and related strain rate obtained in Figure 2 are substituted into Equations (6) and (11). The theoretical stress is compared with the experimental stress, and the comparison results are shown in Figure 4.

The average relative error between the calculated stress value and the measured value is 0.15%. It can be seen that the hyperbolic sine function of the Z parameter can accurately predict the flow stress of 6082 aluminum alloy during high-temperature deformation.

## 3. Establishment of Finite Element Model

### 3.1. Material Model

The material used in this project is 6082 aluminum alloy. After homogenization treatment of the product, the material properties are changed considerably. It is necessary to define the flow stress of 6082 aluminum alloy in the preprocessed attribute library according to constitutive Equation (4) at high temperature, as presented in Section 2. Details of the finite element simulation parameters are shown in Table 2.

### 3.2. D Model

#### 3.2.1. Die Parameter Settings

**Figure 5:**
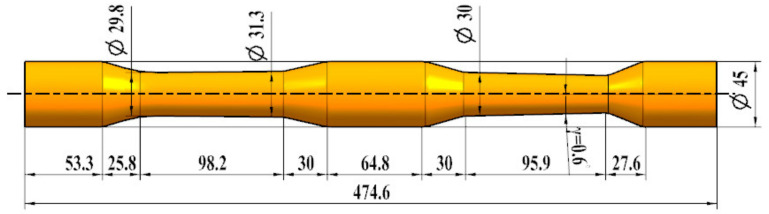
Schematic of the workpiece.

The cross-wedge rolling blank is a 6082 aluminum alloy rod, as shown in Figure 5. The maximum section shrinkage of the cross section is 62.4%, which exceeds the limit of 60.56% for of normal temperature dies. Therefore, in order to reduce the deformation resistance, it is necessary to maintain the forming temperature of the deformation area, and die preheating is used to realize the forming process.

**Figure 6:**
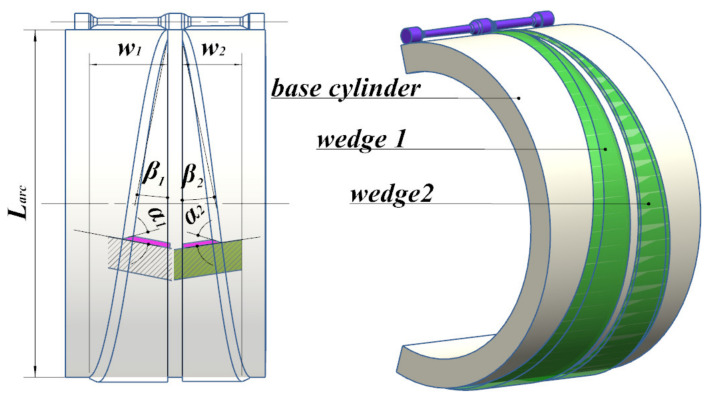
3D mold drawing.

When the section shrinkage is within 60–80%, a forming angle between 18° and 24° should be selected to avoid internal defects. The forming angle, α, is set to 24°. The expansion angle of between 4° and 8° should be selected, with an expansion angle of β1 = 7°, β2 = 6.73° [18]. The corresponding mold design parameters are shown in Table 3, and the 3D mold model established according to the data in Table 3 is shown in Figure 6.

#### 3.2.2. Positioning of the Model

**Figure 7:**
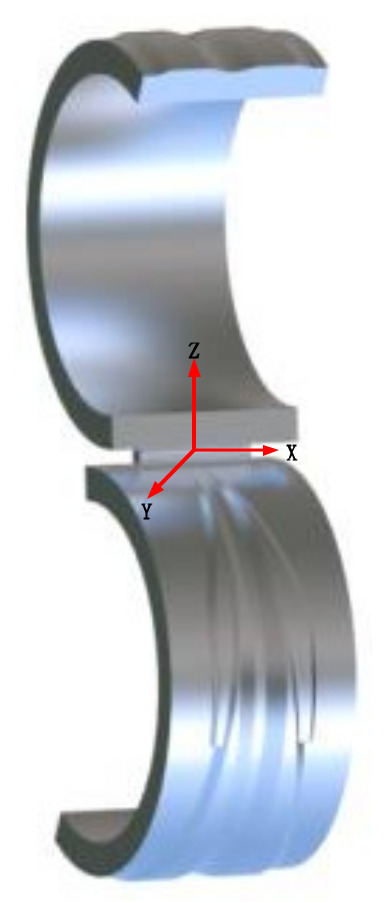
Position of cross-wedge rolling.

Because the modeling function of Deform software is not convenient compared with professional modeling software, the position of the die can be adjusted in UG software first. Then, the division bits are imported into the multi-step simulation module of Deform software. The axis of the cross-wedge rolling piece is set as the X axis, and the center is at the origin of the coordinate. The model and position relationship of the cross-wedge rolling blank step established in UG are shown in Figure 7.

**Figure 8:**
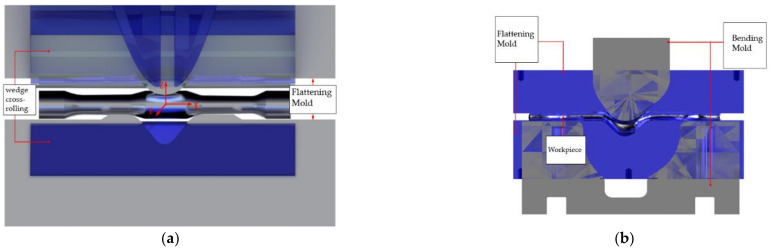

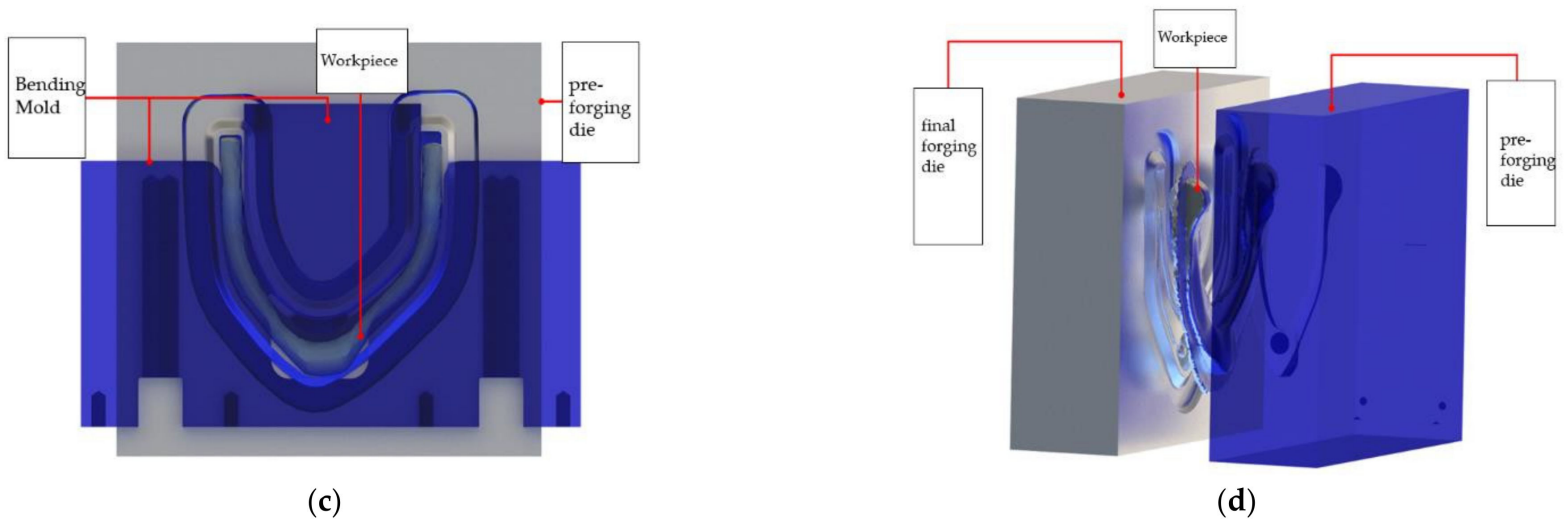
Location of dies. (**a**) Position of flattening die; (**b**) position of bending die; (**c**) position of preforging die; (**d**) location of final forging.

The flattening die is assembled with reference to the cross-wedge rolling position in the first step, taking the rolled workpiece as the center. Theoretically, the position of the workpiece does not change after rolling. The assembly position of the flattening die and the position of the cross-wedge rolling die are shown in Figure 8a.

The initial position of the bending die can be determined by flattening the die and its termination position, as shown in Figure 8b.

After the position of the bending die is set, the inner contour of the preforging die is parallel to the outer contour of the bending die; the specific position is shown in Figure 8c. After bending, the products must be accurately placed into the preforging die.

After the position of the preforging die is fixed, the final position of the final forging die is determined. Because the contour of the two dies is basically parallel, the positioning is easy to set. The specific position is shown in Figure 8d.

After the above steps are completed, according to the simulated multi-step position, the STL file is exported and imported into each preliminary treatment station in the multi-step simulation in Deform software. Then, the preprocessing program of the multi-step simulation is started. After all the steps are set up, the simulation can be carried out. The process can be analyzed in the postprocessing module when the simulation is completed.

### 3.3. Setting Initial Conditions of Finite Element Simulation

The friction model used in the finite element simulation of hot forging is a combination of Coulomb friction and friction law, which is the standard model for forging simulations. In the combined model, the shear stress first increases linearly with an increase in friction coefficient. The shear stress is also limited by the maximum shear yield strength of the friction law [19,20]. The simulation parameters for the forming process are shown in Table 4.

## 4. Forming Process Analysis

### 4.1. Analysis of Blank Preparation Process by Cross-Wedge Rolling

As a key starting step, cross-wedge rolling has a considerable influence on the subsequent process. The forming quality is affected by temperature, rolling speed, die parameters and section shrinkage. The cross-wedge rolling part of this simulation adopts the secondary wedge simulation. The rolling speed is 1 rad/s, the preheating temperature of the rolled piece is 480 °C, the preheating temperature of the die is 200 °C, the die roll gap is 46 mm and the section shrinkage of the rolled piece is 73.4%. Stress is the main factor affecting material deformation. In this paper, we only analyze the stress change in the transition region from the wedge-in section to the widening section because the stress distribution is complex in this region.

**Figure 9:**
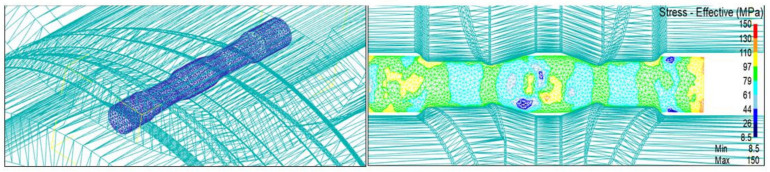
Stress variation in the transition region from the first wedge into the widening section.

Figure 9 shows the variation in stress from the wedge-in section to the widening section. It can be seen from the figure that the stress reaches 110 MPa in the area in contact with the die. The stress in the central region is also relatively high due to the influence of expansion extrusion on both sides of the material. The extrusion stress at the end reaches 150 MPa and is mainly concentrated on the expansion surface, the extrusion surface and the end face of the rolled piece.

**Figure 10:**
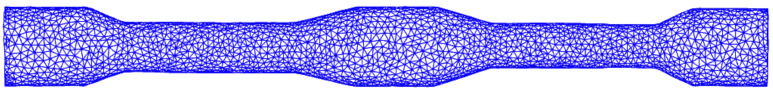
Simulation results of rolled piece.

Figure 10 shows the simulation results. It can be seen that no drawing or necking phenomenon occurs in the rolled piece, nor is there a torsion/distortion cell. The thin rod part maintains a reasonable taper.

### 4.2. Analysis of Filling Process

#### 4.2.1. Comparison of Filling Process

**Figure 11:**
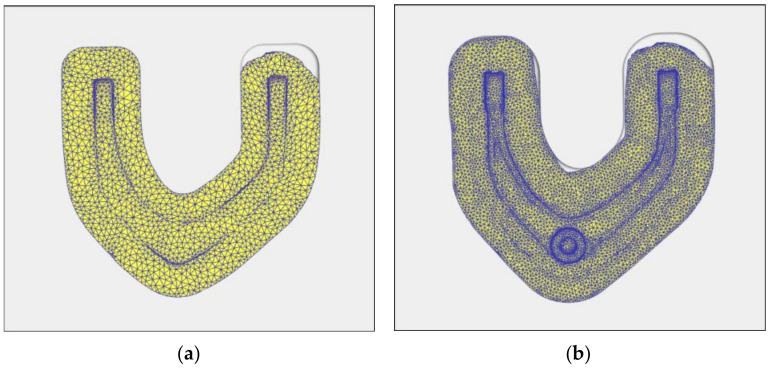
Filling of Forging. (**a**) pre-forged; (**b**) final forging.

In the traditional process, because the maximum demand of each broken face is taken as the benchmark in the blank design, the material is abundant in die forging. As shown in Figure 11, the workpiece and the whole cavity of the die are fully contacted. The final simulation results of the forging show that the contour of the rolled piece is clear, indicating that the product achieves good forming quality.

**Figure 12:**
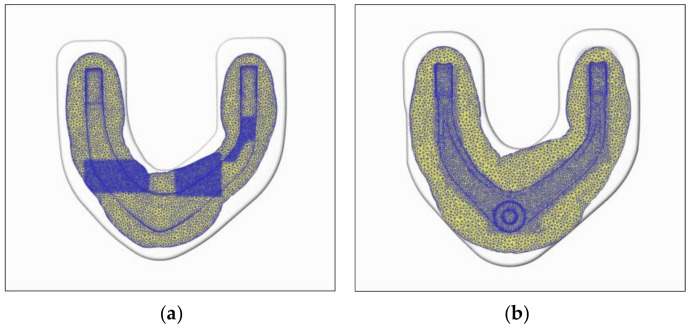
Analysis of preforging forming and final-forging forming. (**a**) Preforging filling; (**b**) final-forging filling.

In the rolling-forging composite forming process, when the whole die side wall contacts the forging blank material, the material is considered to have been filled with cavities. The simulation results of die-forging filling are shown in Figure 12. The blank rolled by cross-wedge rolling is compressed and bent, and then preforging and final forging are carried out. The forming is more sufficient, and the product outline is clear. Because the material distribution is more reasonable, the deformation of the product is more uniform in the forming process.

#### 4.2.2. Comparison of Process Deviation

The preforging and final forging of the forging piece in the traditional process are shown in Figure 11. The profile of the material in each direction exceeds the product profile, and the material is even filled with the flash groove of the cavity. Therefore, sufficient material can ensure the forming accuracy of the product. Whether the blank positioning is correct or a small deviation in the length of the cutting blank will not adversely affect the filling of the forging.

**Figure 13:**
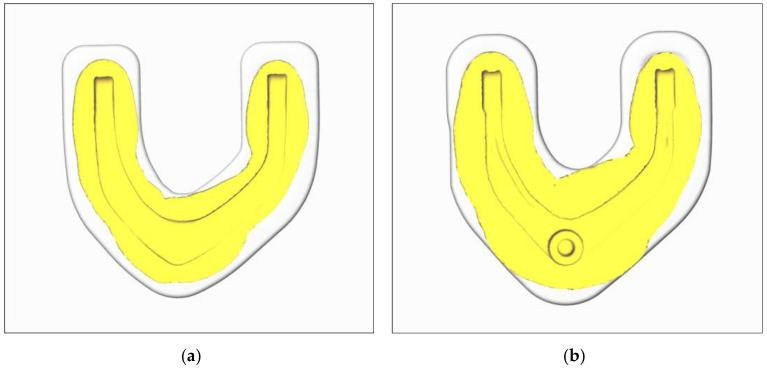
Forming error analysis of preforging and final forging. (**a**) Preforging filling; (**b**) final-forging filling.

In the rolling-forging composite forming process, the simulation results show that the material distribution around the forging contour is uniform, and the forming contour is clear, without defects. The material around on the thin wall and high rib parts is fully formed, and the material in the flash groove exceeds the contour by at least 30 mm, as shown in Figure 13. The filling process can be considered to be sufficiently completed.

#### 4.2.3. Comparison of Forming Force

**Figure 14:**
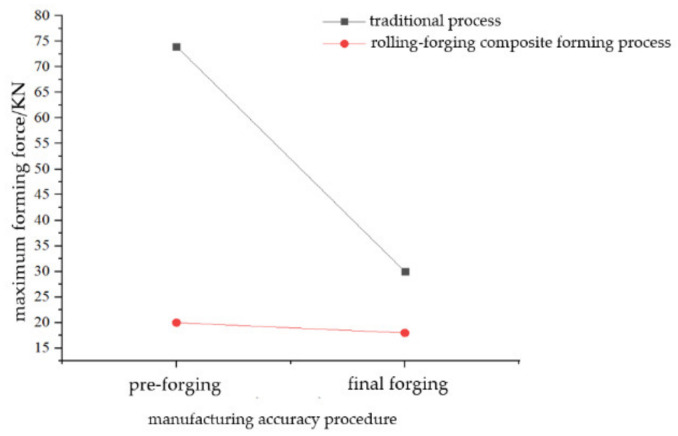
Forging maximum forming force analysis chart of preforging and final forging.

Figure 14 is a maximum forming force analysis diagram of preforging and final forging. In the traditional process, due to the low deformation resistance of aluminum alloy at high temperatures, forging can be realized within 100 kN. The maximum forming forces of preforging and final forging are 74 KN and 30 KN, respectively. In the rolling-forging composite forming process, due to the material redistribution, the waste entering the flash groove is significantly reduced, which significantly reduces the deformation resistance during clamping. As shown in Figure 14, the maximum forming force of preforging is less than 18 KN, and the maximum forming force of final forging is not more than 20 KN. Therefore, it is more energy-saving, and the requirements for equipment can be reduced.

### 4.3. Improvement of Forgings by Rolling-Forging Composite Forming Process

In this section, we will analyze the influence of the rolling-forging composite forming process on product quality according to the three aspects of temperature field, displacement field and strain field after product formation (two steps of preforging and final forging).

#### 4.3.1. Comparison of Temperature Field, Displacement Field and Strain Field in Preforging

**Figure 15:**
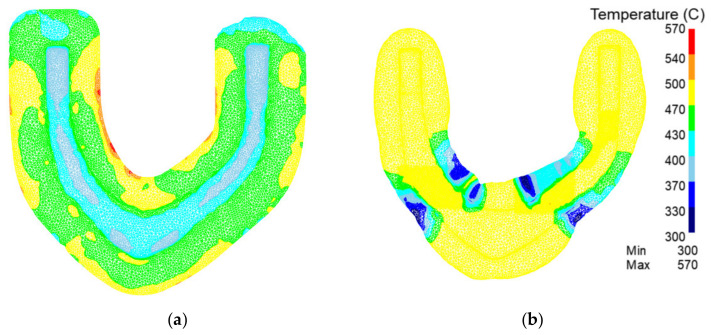
Analysis of preforging temperature field of the traditional process and the rolling-forging composite process. (**a**) Traditional process; (**b**) rolling-forging composite process.

Figure 15 shows a comparison of the temperature fields of forgings with the traditional process vs. the rolling-forging composite process in preforging. In the traditional process, it can be seen that the temperature is obviously low after forming. The main reason is that the flash volume produced in the traditional process is more than that of the rolling-forging composite process, which increases heat conduction. In the rolling-forging composite process, the product temperature is more than 50 °C higher than that of the traditional process, so the deformation resistance of aluminum alloy is lower, and the forming force is smaller. However, there are some low-temperature areas in the rolling-forging composite process. This is because due to the existence of deformation work, the temperature rise is more obvious in the area where the material is most deformed. The roll-forging composite process redistributes the volume of the billet, resulting in minimal deformation.

**Figure 16:**
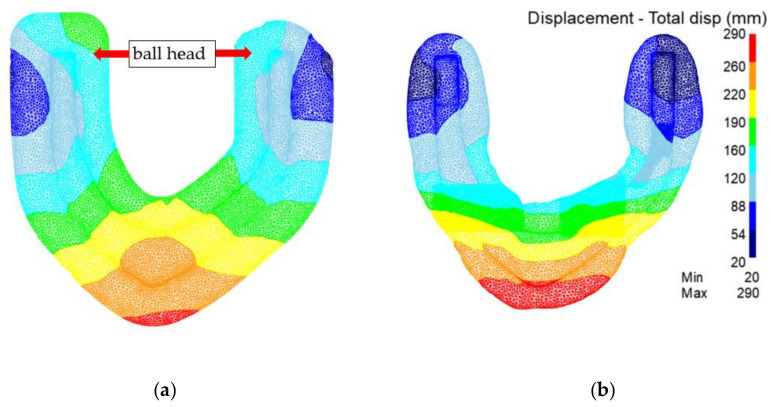
Analysis of displacement field in preforging of the traditional process and the rolling-forging composite process. (**a**) Traditional process; (**b**) rolling-forging composite process.

Figure 16 is the displacement field of the preforging process. It can be seen that the metal flow range of the traditional process is relatively large. The metal flow area of the rolling-forging composite process is small, and the ‘ball head’ part is a slow flow area. It can be seen that the forming difficulty of the product is significantly reduced after the material is redistributed by the rolling-forging composite process.

**Figure 17:**
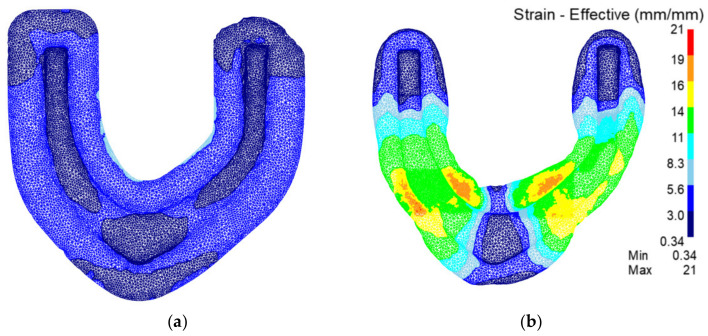
Analysis of preforging strain field of the traditional process and the rolling-forging composite process. (**a**) Traditional process; (**b**) rolling-forging composite process.

Figure 17 shows the strain field of the preforging process. It can be seen that the strain in the traditional process is significantly lower than that in the rolling-forging composite process. In products made with the rolling-forging composite process, the deformation of the whole contour is more sufficient and uniform. Therefore, there are fewer internal defects after the rolling-forging composite process.

#### 4.3.2. Comparison of Temperature Field, Displacement Field and Strain Field in Final Forging

**Figure 18:**
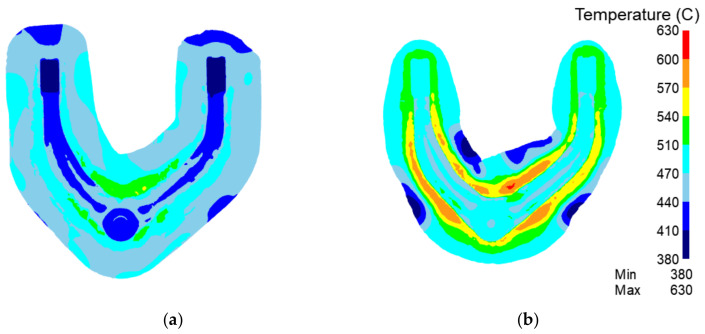
Analysis of final forging temperature field of the traditional process and the roll-forging composite process. (**a**) Traditional process; (**b**) rolling-forging composite process.

Figure 18 shows the temperature field of the final forging process. It can be seen that the regions with high temperature are all at the edge of the product contour. In the rolling-forging composite process, the temperature is higher than that of the traditional process, which is beneficial to the metal flow. The high-temperature zone of the contour edge of the rolled piece reaches more than 570 °C. The deformation resistance of the metal is low, and the metal is more likely to flow out, so the forging has higher uniformity in the forming process.

**Figure 19:**
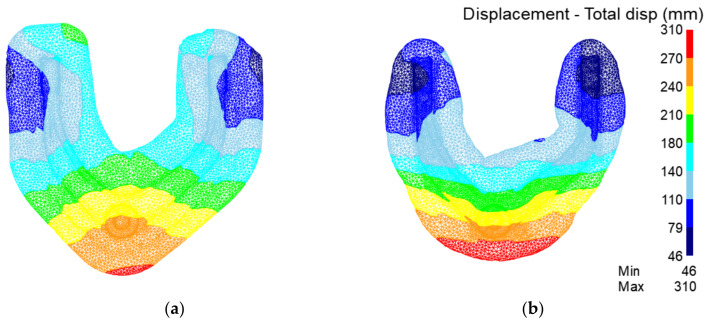
Analysis of displacement field in the final forging of traditional process and the rolling-forging composite process. (**a**) Traditional process; (**b**) rolling-forging composite process.

Figure 19 shows a comparison of the displacement fields of the final forging process. It can be seen that the metal flow range of the traditional process is relatively large. The metal flow range of the rolling-forging composite process is small, and the ‘ball head’ part is a slow flow area, so the distortion energy of metal storage in the traditional process is higher. The metallographic structure is also more prone to a coarse-grain structure.

**Figure 20:**
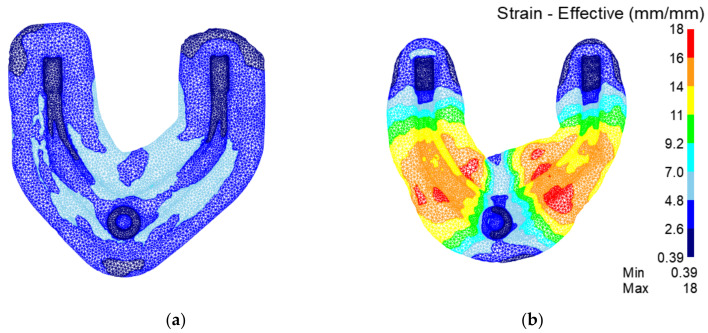
Analysis of final forging strain field of the traditional process and the roll-forging composite process. (**a**) Traditional process; (**b**) rolling-forging composite process.

Figure 20 shows the strain field of the final forging process. It can be seen that the strain in the traditional process is significantly lower than that in the rolling-forging composite process. The deformation of rolling-forging composite process products in the whole contour is more sufficient. Therefore, the forming effect of the rolling-forging composite process is obviously better than that of the traditional process.

## 5. Quality Control of Rolling-Forging Composite Forming Process

The rear upper control arm of an automobile is a complex ribbed part, and its forming process is very complex. As an automobile part, it has high requirements not only for the shape of the product but also for the internal structure and grain size of the product. In order to control the quality and performance of products, the raw materials must be homogenized, the internal structure of aluminum alloy bars must be uniform and the grains must reach first-grade size. Due to the deformation and uneven distribution of the temperature field in the product forming process, a large amount of distortion energy is accumulated to produce a coarse-grain structure, which seriously weakens the product performance. The optimal process parameters to ensure product quality are determined by analyzing the coarse-grain structure at different process temperatures and heat treatment temperatures.

### 5.1. Analysis of the Location of Coarse-Grain Structure

When the workpiece goes through multiple thermal deformation processes, such as cross-wedge rolling of the billet, flattening, bending, preforging, final forging, solid solution, etc., it produces a large number of uneven deformations. Because an uneven, large deformation has an important influence on the formation and changes the coarse-grain zone, it is important to analyze and study the thermal deformation process of all processes for product quality control. The coarse grain structure of aluminum alloy die-forging products is generally formed in the region with the largest deformation.

**Figure 21:**
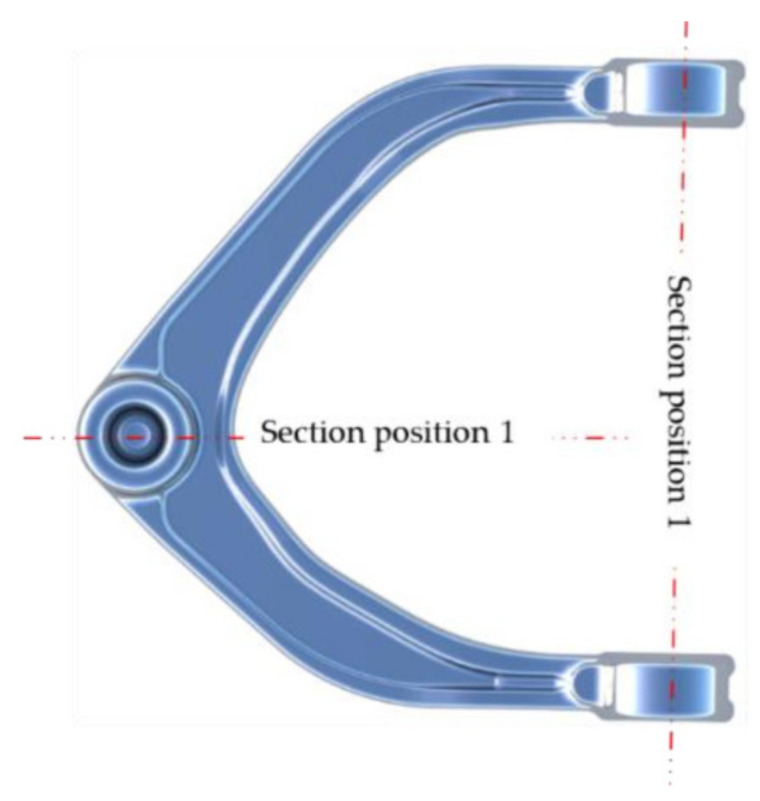
Cutting position.

As shown in Figure 21, during the whole deformation process, ‘ring bulge’ position 1 and ‘ball head’ position 2 are the largest deformation positions, where a coarse grain structure is prone to appear.

### 5.2. Effect of Material Temperature on Coarse-Grain Structure

Under the same conditions, the preheating temperature of the material has a significant effect on the coarse-grain structure on the product surface. Within a certain temperature range, with an increase in temperature, the deformation resistance of the metal decreases, and the shear stress of the die side wall on the metal also decreases, which reduces the distortion energy of the metal. Therefore, the thickness of the coarse-grain area on the surface decreases. When the temperature exceeds a certain range, the higher the energy stored on the product surface, the greater the power provided for recrystallization due to the high surface temperature of the material. Hence, it is prone to produce coarse-grain defects.

**Figure 22:**
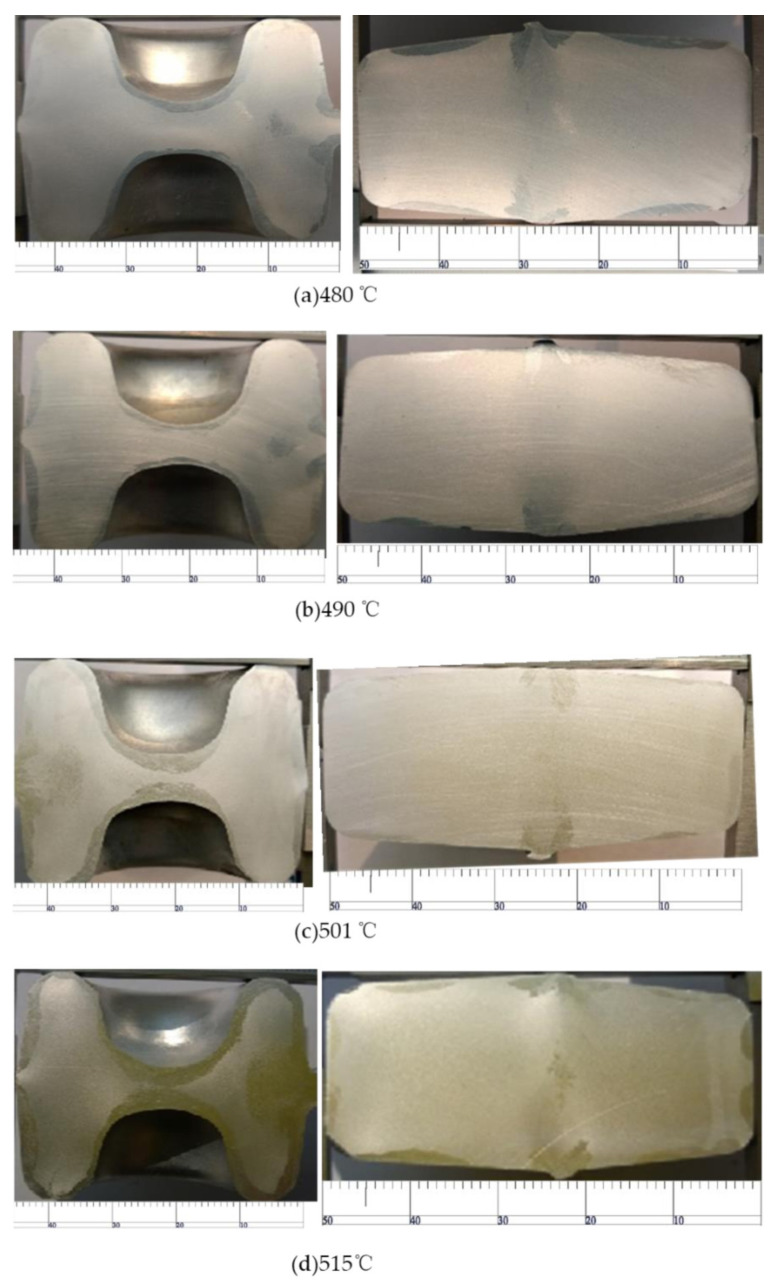
Coarse-grain structure of material at different preheating temperatures.

As shown in Figure 22, when the preheating temperature of the material increases from 480 °C to 490 °C, the coarse-grain microstructural layer on the surface becomes significantly thinner. When the temperature increases from 490 °C to 501 °C, the temperature rise of the material provides more power for the recrystallization of the aluminum alloy, which makes the coarse-grain microstructure on the surface thicker. Even up to 515 °C, the coarse-grain structure at the thin wall almost runs through the whole wall, which seriously reduces the mechanical properties of the material.

### 5.3. Effect of Flattening and Bending Temperature on Coarse-Grain Structure

**Figure 23:**
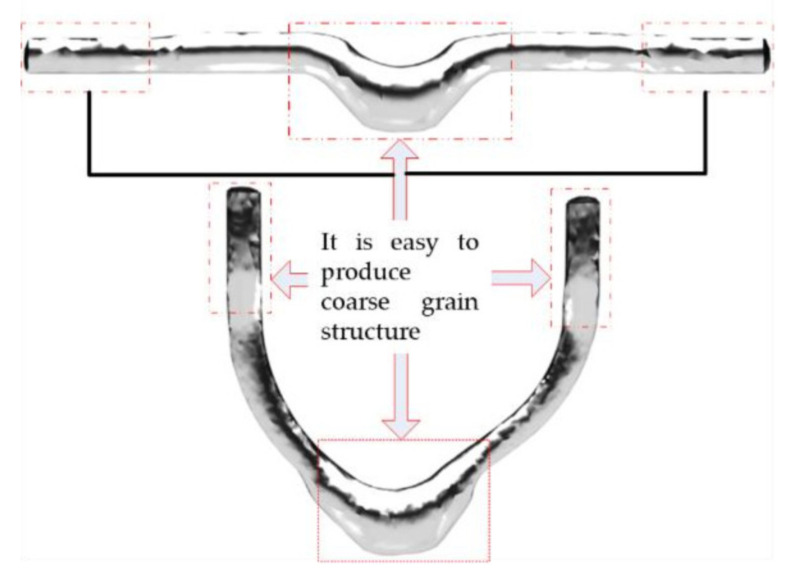
The position where a coarse-grain structure is easily produced by the bending process.

Due to the considerable deformation of both ends in the flattening process and the subsequent preforging process, a large amount of distortion energy is stored, and a coarse-grain structure is easily produced. The bending process has a considerable influence on the contour shape of the product. As shown in Figure 23, the shape of the center position changes considerably in during bending deformation, which has a considerable influence on the subsequent process. The preheating temperature of the flattening and bending die affects the coarse-grain structure of the product.

**Figure 24:**
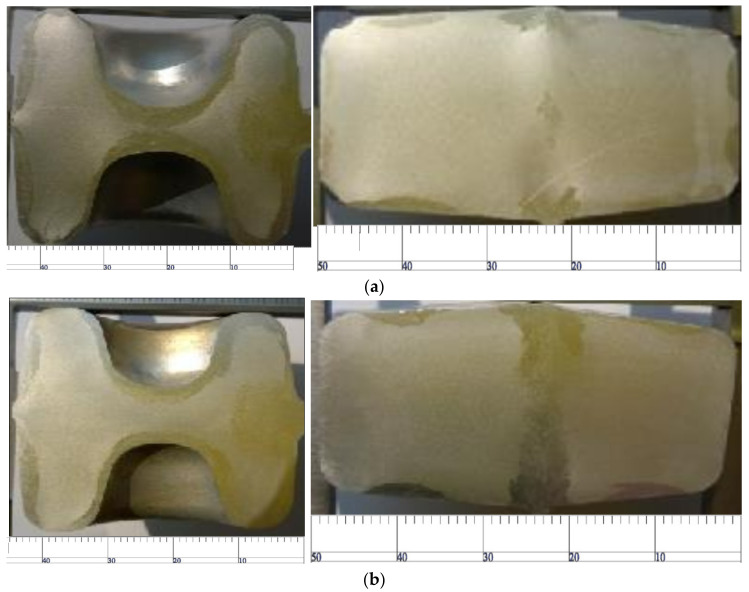
Effect of heating temperature of the bending process die on the coarse-grain structure. (**a**) Flattening preheating temperature of 378 °C/bending preheating temperature of 300 °C; (**b**) flattening preheating temperature of 400 °C/bending preheating temperature of 340 °C.

As shown in Figure 24, due to the insufficient preheating temperature of the die, the friction between the die and the material surface causes the recrystallization of the material. Increasing the process temperature can effectively reduce the deformation resistance of the material, so increasing the preheating temperature of the flattening and bending dies can significantly improve the coarse-grain structure of the rolled piece. For the ‘ball head’ part, due to almost no deformation during bending, the preheating temperature has little effect on the coarse-grain structure.

### 5.4. Effect of Secondary Heating on Coarse-Grain Structure

**Figure 25:**
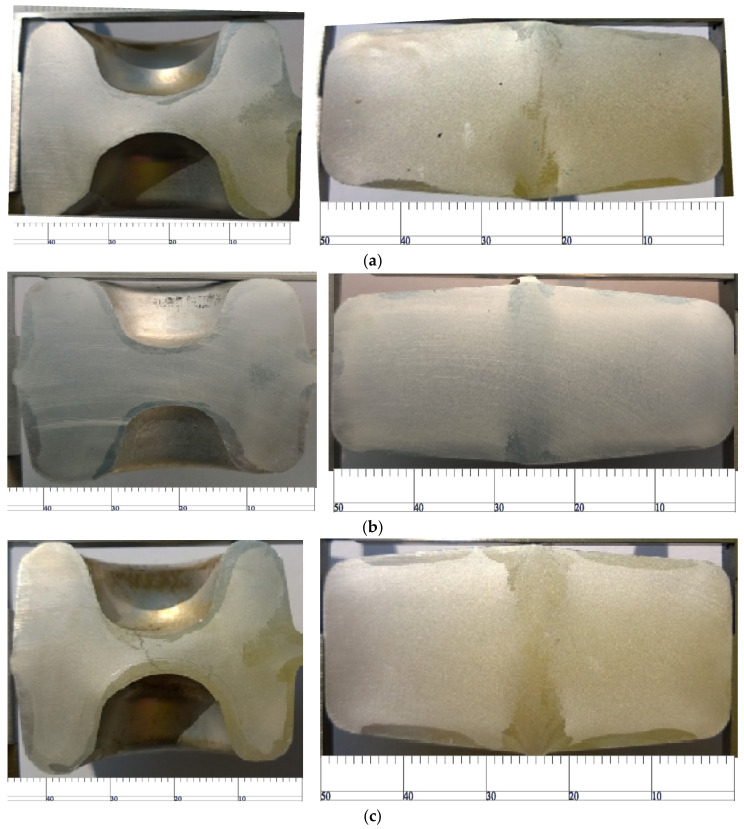
Effect of secondary heating on coarse-grained structure. (**a**) 490 ℃; (**b**) 500 ℃; (**c**) 510 ℃.

In order to improve the forming performance, the product often requires secondary heating in the intermediate process. Under the same temperature as the other processes, secondary heating has a significant impact on the coarse-grain structure. As shown in Figure 25, when the secondary heating of the workpiece after bending increases from 490 °C to 500 °C, the thickness of the coarse-grain layer decreases significantly. When the temperature rises from 500 °C to 510 °C, the coarse-grain structure of both the high convex part and the ball head position increase obviously, and the thickness of the high convex part increases obviously. In summary, a secondary heating temperature of 500 °C is most favorable for product performance.

### 5.5. Effect of Die Forging Temperature on Coarse-Grain Structure after Forging

**Figure 26:**
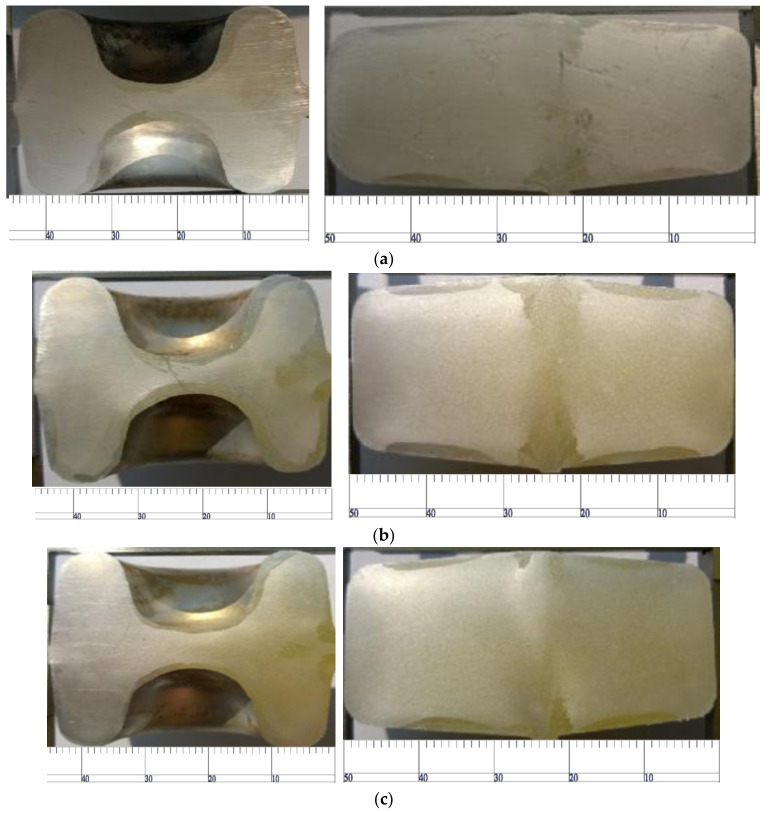
Effect of die preheating temperature on coarse-grain defects in the die forging process. (**a**) Preheating temperature of upper die is 323 °C; preheating temperature of lower die is 322 °C. (**b**) Preheating temperature of upper die is 335 °C; preheating temperature of lower die is 340 °C. (**c**) Preheating temperature of upper die is 350 °C; preheating temperature of lower die is 350 °C.

Die forging temperature is the process parameter that has the greatest influence on the coarse-grain structure. In order to observe the influence of die temperature on the coarse-grain structure of the rolled piece, the experimental conditions are set as follows: the measured preheating temperature of the billet is 480 °C, solid solution treatment at 500 °C for 50 min, aging temperature of 170 °C and aging time of 540 min. The preheating temperatures of the upper and lower dies are shown in Figure 26.

As shown in Figure 26a, when the preheating temperature of the upper die is 323 °C and the preheating temperature of the lower die is 322 °C, the thickness of the coarse-grain layer is about 3 mm and uniform. As shown in Figure 26b, when the preheating temperature of the upper die is 335 °C and the preheating temperature of the lower die is 340 °C, the thickness of the coarse-grain layer in the upper part is only 1.5 mm, whereas that in the lower part is 3.5 mm. As shown in Figure 26c, the temperature of the upper and lower die rises to 350 °C, the coarse grain structure of the contact part of the upper and lower die decreases and the layer thickness is only 1.5–2 mm. This is because the increase in temperature reduces the deformation resistance of the material, so the deformation of the surface metal is reduced because of the shear force, which improves the coarse-grain structure of the material. Therefore, 350 °C is a good process temperature.

### 5.6. Effect of Solution Treatment on Coarse-Grain Structure after Die Forging

For most non-ferrous alloys, the purpose of solid-solution treatment is to obtain supersaturated solid solution and produce precipitates for subsequent aging treatment. Main parameters of solid-solution treatment include heating temperature, holding time and cooling rate. The upper-limit temperature of heating temperature is usually close to the solid-line temperature or eutectic temperature. At this high temperature, the alloy has the maximum solid solubility and a fast diffusion rate. However, the temperature should not be too high; otherwise, it will lead to an overburning phenomenon. The minimum heating temperature should be higher than the solid solubility curve; otherwise, the performance after aging cannot meet the requirements. Based on this, the solid solution temperature of the billet is set to 530–540 °C, with a solid solution time of 30–45 min.

**Figure 27:**
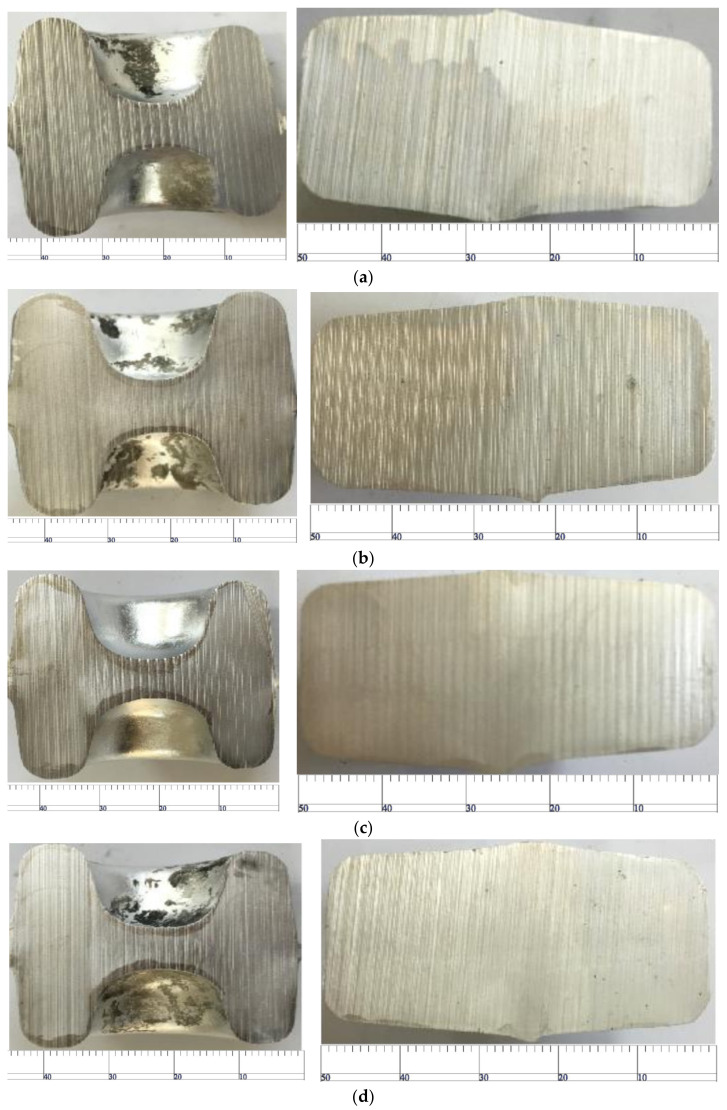
Effect of solution treatment on coarse-grain structure after die forging. (**a**) Solution temperature: 530 °C, solution time: 30 min; (**b**) solution temperature: 530 °C, solution time: 45 min; (**c**) solution temperature: 540 °C, solution time: 30 min; (**d**) solution temperature: 540 °C, solution time: 45 min.

As shown in Figure 27a,b, when the preheating temperature of the product is 530 °C, the precipitated phase in the forging prevents the surface grain from growing. With the increase in time, the thickness of the coarse-grain layer decreases significantly. As shown in c,d, as the temperature continues to rise, the precipitates begin to dissolve, and the surface grains tend to grow, resulting in a thicker coarse-grain layer. Therefore, it can be inferred that the optimal process scheme is achieved when the preheating temperature is 530 °C and the solid solution time is 45 min.

## 6. Conclusions

1. The proposed rolling-forging composite process can greatly reduce the material cost and reduce the forming force, resulting in superior product performance. In the rolling-forging composite process, the cross-wedge rolling process is the core of the whole process and guarantees collaborative control of the product shape.
2. The temperature, time of process and heat treatment have an important influence on the coarse-grain structure of the product, and the coarse grain structure of the rear upper control arm of the automobile is mainly concentrated in the maximum deformation parts of the product, namely the ‘ring bulge’ and ‘ball head’ positions. The optimal preheating temperature for die forging is 350 °C. A preheating temperature of 530 °C and a solution time of 45 min results in the least coarse-grain surface structure.
3. The results of this study provide a theoretical basis for improving the quality of multi-step forming of automobile rear upper control arms.

## Figures and Tables

**Table 1:** Peak stress of 6082 aluminum alloy at different temperatures and strain rates.

Temperature	Strain Rate	Peak Stress
350 ℃	0.01	42.75
	0.10	32.17
	1.00	25.72
	5.00	20.70
400 ℃	0.01	52.90
	0.10	40.35
	1.00	32.26
	5.00	28.04
450 ℃	0.01	68.68
	0.10	51.63
	1.00	43.24
	5.00	36.34
500 ℃	0.01	78.72
	0.10	61.28
	1.00	51.14
	5.00	49.38

**Table 2:** Simulation parameters for cross-wedge rolling.

Parameter	Numerical Value
Angular velocity (rad/s)	0.6/0.8/1
Preheating temperature of workpiece (T0) (°C)	500
Ambient temperature (°C)	20
Heat transfer coefficient (N/s/mm/°C)	75
Mixed heat dissipation coefficient, q (N/s/mm/°C)	0.008 Tb
Friction axis factor	1.5
Number of deformable grids	20,000
Base diameter (mm)	670
Metamorphic materials	6082 aluminum alloy

**Table 3:** Design parameters of molds.

Number	Roller Radius (mm)	Wedge Width w(mm)	Forming Angle α(°)	Expansion Angle β(°)
wedge 1	480.5	182.4	24	7
wedge 2	480.5	175.3	24	6.73

**Table 4:** Simulation parameters for the forming process.

Project		Parameter	Remark
Aluminum billet material		6082 aluminum alloy	
Preheating temperature of billet		500	
Speed of hydraulic press (mm/s)		250	
Initial billet temperature, Tb (°C)		500	
Initial mold temperature. Td (°C)		350	
Ambient temperature (°C)		30	
Coulomb friction coefficient		0.3	
Shear friction coefficient		1.5	
Heat transfer coefficient (N/s/mm/°C)		75	
Mixed cooling coefficient of radiation convection (N/s/mm/°C)	Cross-wedge rolling	4	Default unit of Deform: N/s/mm/°C
	Collapsing	4	
	Bending	4	
	Preforging	0.02	
	Final forging	0.02	

## References

[1] Wang W. (2018). Present situation and development trend of automotive materials. New Mater. Ind..

[2] Zhang Y. (2017). Application of automobile lightweight and aluminum alloy in modern automobile production. Intern. Combust. Engines Accessories.

[3] Xie Y. Application and Development of Aluminum Alloys in the Automotive Industry. Proceedings of the Annual Conference of China Aluminum Processing Industry in 2018.

[4] Zhou J., Shi P.C., Li Y.Z. (2017). Simulation analysis of forging process of automobile aluminum alloy swing arm. J. Xinxiang Univ..

[5] Zhao P.F. (2006). Study on Cross Wedge Rolling Deformation of 6061 Aluminum Alloy. Ph.D. Thesis.

[6] Wang G.B. (2018). Automobile aluminum alloy control arm forging hydraulic unit. Mech. Des..

[7] Bao Q.H., Pan Q.J., Wu S.X., Cheng J. (2011). Die forging forming of automobile aluminum alloy control arm. Met. Processing.

[8] Wei W. (2013). Hot Deformation Behavior and Microstructure and Properties of 6082 Aluminum Alloy Rib Forgings. Ph.D. Thesis.

[9] Su H.Y. (2005). Research and development of aluminum for automobile industry. Nonferrous Met. Ind..

[10] Li X.D., Shi W.C., Zhang H., Xue K.M. (2011). Die forging process design and numerical simulation of aluminum alloy control arm. Die Technol..

[11] Sellars C.M., Mctegart W.J. (1966). On the mechanism of hot deformation. Acta Metall..

[12] Shi H., Mclaren A.J., Sellars C.M., Shahani R., Bolingbroke R. (1997). Constitutive equations for high temperature flow stress of aluminium alloys. Mater. Sci. Technol..

[13] Zener C., Hollomon J.H. (1944). Effect of Strain Rate Upon Plastic Flow of Steel. J. Appl. Phys..

[14] Jia Y.J. (2013). Experimental Study and Numerical Simulation of Thermal Deformation and Dynamic Recrystallization Behavior of 7050 Aluminum Alloy. Master’s Thesis.

[15] Estrin Y., Tóth L., Molinari A., Bréchet Y. (1998). A dislocation-based model for all hardening stages in large strain deformation. Acta Mater..

[16] Grong O., Shercliff H.R. (2002). Microstructural modelling in metals processing. Prog. Mater. Sci..

[17] Lee Y.C., Hwang E., Shih Y.P. (1994). A combined approach to fuzzy model identification. Syst. Man Cybern. IEEE Trans..

[18] Hu Z.H., Zhang K.S., Wang B.Y. (1996). Cross Wedge Rolling Theory and Application.

[19] Altan T., Vazquez V. (1996). Numerical Process Simulation for Tool and Process Design in Bulk Metal Forming. CIRP Ann. Manuf. Technol..

[20] Behrens B.A., Bouguecha A., Hadifi T., Mielke J. (2011). Advanced friction modeling for bulk metal forming processes. Prod. Eng..

